# The capping arms race: evolutionary interplay between IFIT proteins and viral molecular mimicry

**DOI:** 10.1007/s11033-026-12001-8

**Published:** 2026-05-22

**Authors:** Saul González-Hilario, Paulina Zepeda-Cendejas, Marcela Esmeralda Cibrian-Segura, Rodolfo Gamaliel Avila-Bonilla

**Affiliations:** Centro de Investigación y de Estudios Avanzados del Instituto Politécnico Nacional, Departamento de Genética y Biología Molecular, Av. IPN 2508, Mexico City, Mexico

**Keywords:** IFIT proteins, viral RNA capping, innate immunity, 2′-O-methylation, antiviral therapeutics

## Abstract

Effective host survival during viral infection depends on the ability to discriminate endogenous messenger RNA from pathogenic RNA species. Interferon-induced proteins with tetratricopeptide repeats (IFITs) constitute a central component of this defence system, functioning as specialised cytosolic RNA surveillance proteins that detect molecular features characteristic of non-self transcripts. This review examines the dynamic evolutionary interplay between IFIT-mediated antiviral restriction and viral evasion strategies. IFIT proteins recognise defined chemical and structural determinants at the RNA 5′ terminus—including 5′-triphosphorylated ends and the absence of 2′-O-methylation—and sequester such transcripts to inhibit viral translation. In response, RNA viruses have evolved sophisticated evasion strategies, including the encoding or acquisition of 2′-O-methyltransferases, exploitation of host cap-modifying enzymes, and cap-snatching mechanisms that generate host-like cap 1 and m⁶Am signatures to evade immune recognition. Beyond the canonical antiviral roles attributed to IFIT1 and IFIT5, accumulating evidence indicates that IFIT2 and IFIT3 function as critical regulatory components within IFIT complexes, stabilising protein–protein interactions, modulating RNA-binding affinity, and influencing downstream signalling pathways. Collectively, these concepts position IFIT proteins as RNA surveillance proteins and regulatory scaffolds within innate immunity. We propose that future antiviral strategies should focus on unmasking viral RNA, thereby restoring its detection by the host innate immune system.

## Introduction

Type I interferons (IFNs) primarily mediate the mammalian innate immune response, inducing a broad repertoire of interferon-stimulated genes (ISGs) that collectively establish a rapid antiviral state and initiate inflammatory signalling pathways. This defence is triggered when pathogen-associated molecular patterns (PAMPs)—highly conserved microbial signatures—are detected by pattern recognition receptors (PRRs). Viral nucleic acids are sensed by multiple PPR families, including Toll-like receptors (TLRs) and retinoic acid-inducible gene I (RIG-I)-like receptors (RLRs). TLRs reside at the cell surface or within endosomal compartments, where they survey extracellular and endosomal environments for pathogenic signatures. Conversely, RLRs—such as RIG-I, melanoma differentiation-associated protein 5 (MDA5), and laboratory of genetics and physiology 2 (LGP2)—act as key mediators of intracellular surveillance by detecting viral RNA species within the cytosol [1–3].

Viral RNAs differentiate themselves from host transcripts through specific features such as long and short double-stranded RNA (dsRNA) bearing 5′-di-/triphosphate termini (5′-pp or 5′-ppp), which serve as ligands for PRRs. Upon ligand engagement, both TLRs and RLRs activate downstream signalling pathways that converge on transcription factors such as interferon regulatory factors (IRFs) and nuclear factor κB (NF-κB), ultimately driving the production of type I interferons and pro-inflammatory cytokines. This coordinated response is essential for mounting an effective early antiviral and inflammatory defence mediated predominantly by ISGs and inflammatory mediators [4, 5].

**Fig. 1:**
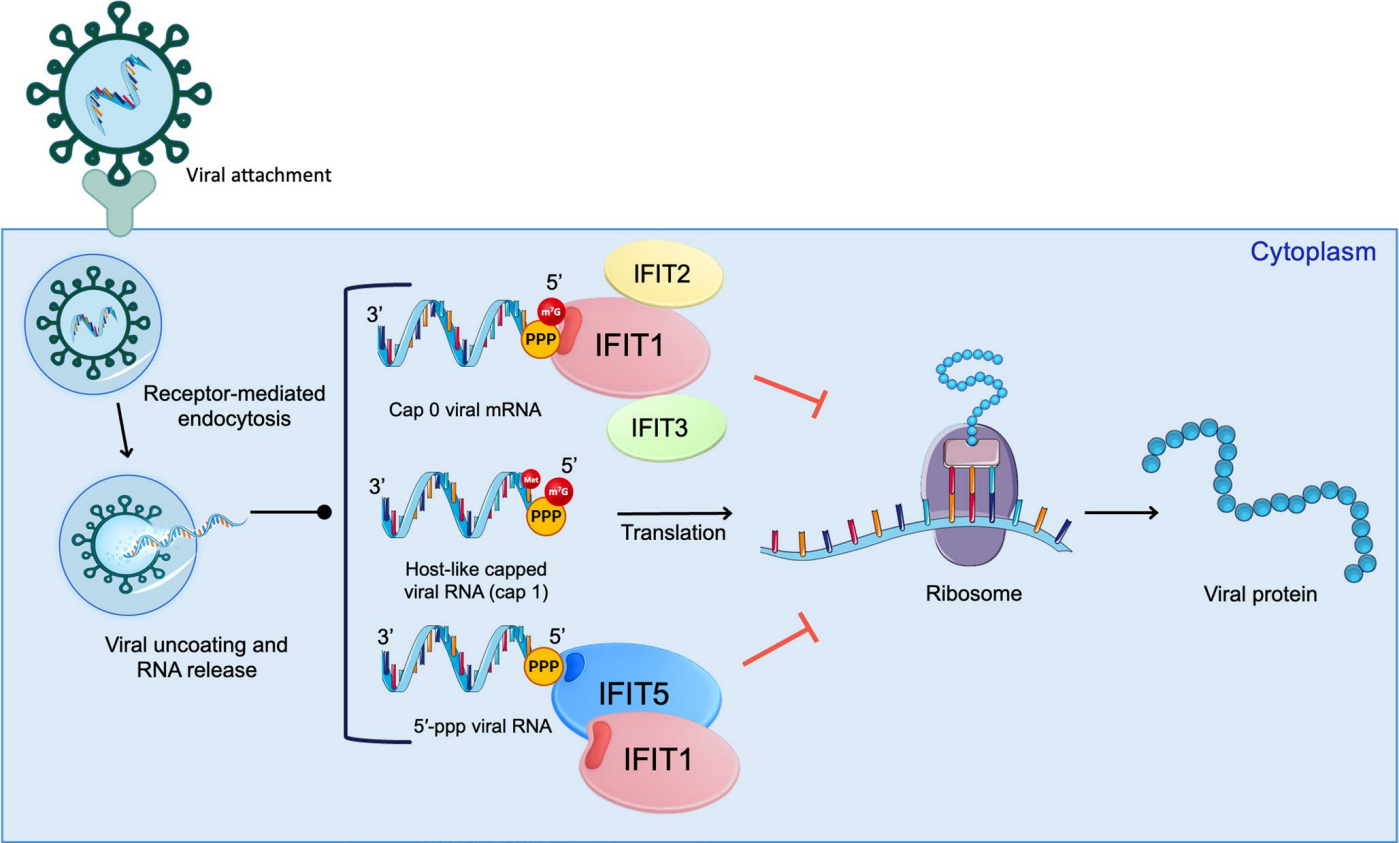
Mechanism of viral RNA recognition by IFIT proteins in the cytoplasm. Upon viral attachment to cell surface receptors, the virus enters via receptor-mediated endocytosis. Following uncoating and the release of the viral genome, three distinct species of viral RNA are illustrated with specific 5′-end configurations, including cap 0, host-like cap 1, and uncapped 5′-ppp RNA. These structures are presented to the host innate immune machinery and dictate selective recognition by members of the IFIT protein family. The IFIT1–IFIT2–IFIT3 complex enhances the stability and specificity of this restriction, preferentially interacting with cap 0 viral RNA to repress translation. Conversely, cap 1 viral RNA—generated through virus-encoded capping machinery—acquires host-like features that allow escape from IFIT1-mediated restriction in most cases, thereby supporting efficient viral protein synthesis and replication. Finally, while both proteins contribute to the detection of uncapped RNA, IFIT5 preferentially binds and sequesters 5′-ppp signatures present on viral genomic RNA and subgenomic RNA products, with IFIT1 playing a secondary role in this recognition

The ISGs leads to the synthesis of a wide range of antiviral proteins that play a crucial role in defending the host against viral infections. These ISG-encoded proteins disrupt various stages of the viral life cycle, including viral entry, genome replication, protein translation, and viral release [6, 7]. Notably, certain ISGs exert direct antiviral effects by interacting with RNA viral genomes, promoting processes such as editing, degradation, or translational arrest. These proteins, which link RNA sensing to innate immune effector functions, are classified as RNA-binding proteins (RBPs). Well-characterised ISGs include ADAR1 (adenosine deaminase acting on RNA), OAS family proteins (2’,5’-oligoadenylate synthetases), PKR (protein kinase R), as well as members of the IFIT (interferon-induced proteins with tetratricopeptide repeats) family [8]. Among these, IFIT proteins restrict replication by sequestering viral 5′ cap structures bearing 5′-ppp groups or lacking 2′-O-methylation, thereby competitively inhibiting eIF4E/eIF4F recruitment and blocking ribosomal translation (Fig. 1) [9–11].

Because the chemical composition of the RNA 5′ terminus constitutes a critical molecular signature for self–non-self-discrimination, viruses and host cells are engaged in a continuous evolutionary arms race centred on this region. It is important to note that DNA viruses, which predominantly replicate within the nucleus, function as molecular parasites of the host transcriptional machinery. By utilising cellular RNA polymerase II and the nuclear RNA capping apparatus, these viruses inherently acquire host-like 5′-end modifications, largely escaping innate immune recognition [5, 8, 9].

In contrast, RNA viruses replicate in the cytoplasm and must encode their own capping enzymes or employ alternative strategies, such as cap-snatching, placing them under strong evolutionary pressure to mimic host RNA structures [12, 13]. In this context, IFIT proteins act as central antiviral effectors by detecting viral RNAs that lack host-specific 5′-end modifications. Accordingly, this review focuses on the molecular mechanisms underlying IFIT-dependent antiviral activity, as well as on the viral RNA capping strategies that enable evasion of IFIT-mediated restriction.

## The IFIT family

The IFIT gene family exhibits a conserved genomic architecture, typically comprising two exons, with the second exon encoding nearly the entire open reading frame. Despite this structural consistency, the organisation of IFIT loci differs markedly among species, likely reflecting divergent evolutionary pressures imposed by co-evolving viruses. Homologues have been identified not only in mammals but also across other vertebrates, including fishes, amphibians, and birds, highlighting their broad evolutionary conservation [14, 15].

### Species-specific organisation

In humans, four well-characterised members of this family—*IFIT1* (ISG56), *IFIT2* (ISG54), *IFIT3* (ISG60), and *IFIT5* (ISG58)—are clustered on chromosome 10. Additionally, the human genome contains an uncharacterised gene, *IFIT1B*, and a pseudogene, *IFIT1P1*, located on chromosome 13. Notably, *IFIT1B* lacks the canonical interferon-stimulated response element (ISRE) found in the promoters of other family members, rendering its expression independent of interferon signalling [16, 17].

In contrast, the murine genome on chromosome 19 contains six *Ifit* genes—*Ifit1*, *Ifit1b*, *Ifit1c*, *Ifit2*, *Ifit3*, and *Ifit3b*—but lacks a functional orthologue of IFIT5, which appears to be conserved across most rodents. Conversely, in birds, the majority of IFIT genes have been lost; however, IFIT5 is retained and appears to preserve functions analogous to those of its human counterpart. Sequence analysis reveals that human and mouse orthologues share 52–62% identity, whereas orthologues from other species display lower similarity (approximately 40–45%), suggesting that the human and mouse repertoires arose through duplication events from a shared ancestral gene [16–19].

### Mechanisms of genetic innovation

A common mechanism of genetic innovation in host genomes is gene duplication, which can provide an avenue to evade viral antagonism or promote the emergence of novel inhibitory functions through subfunctionalisation. In the case of IFIT genes, variation arising from such duplication events across species reflects the adaptation of viral restriction factors with species-specific antiviral activities, thereby sustaining effective defences against a constantly evolving array of pathogens. For instance, in mice, the duplication of an ancestral gene has led to paralogues with distinct functions. While mouse *Ifit1b* has the capability to restrict the translation of mRNAs lacking a 5′-cap structures, mouse *Ifit1* does not possess this function [10, 13]. This contrasts with human IFIT1, which also restricts such mRNAs.

Functional divergence is also evident even among closely related species, such as humans and chimpanzees, particularly regarding their capacity to restrict Venezuelan equine encephalitis virus (VEEV). This variation has been mapped to just two amino acid substitutions within α-helix 18 of the tetratricopeptide repeat (TPR) 7 domain. Whereas the chimpanzee IFIT1 variants at these positions render the protein permissive to VEEV replication, the corresponding human residues act as key determinants of IFIT1’s potent antiviral activity [10]. This evolutionary perspective sets the stage for a detailed examination of IFIT protein structures, providing critical insights into the structural basis of their antiviral mechanisms and functional diversity.

### The biochemistry of 5′-end recognition

**Fig. 2:**
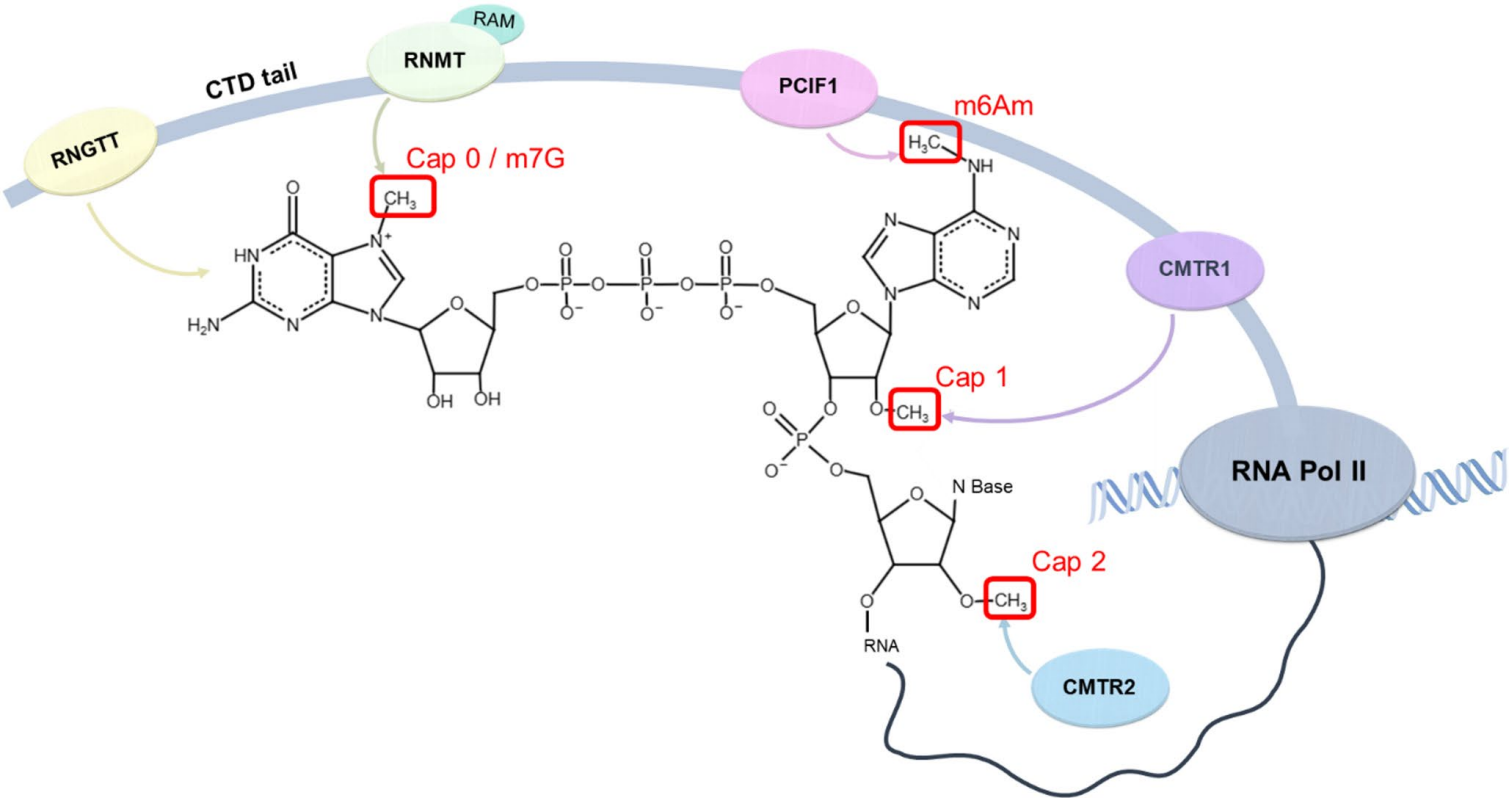
Co-transcriptional capping and methylation of mammalian mRNA. The formation of the 5′ cap is initiated co-transcriptionally by the recruitment of capping enzymes to the phosphorylated C-terminal domain (CTD) of RNA Pol II. The enzyme RNGTT acts on the nascent transcript to form the unmethylated G-cap (cap 0 precursor). Subsequent methylation steps convert this precursor into mature cap structures. RNMT, in complex with its activating subunit RAM, methylates the guanosine cap at the N7 position to form cap 0 (m⁷G cap). Further modifications occur on the first and second transcribed nucleotides to generate self-identifying signatures. CMTR1 adds a methyl group to the 2′-O position of the first nucleotide, yielding cap 1 structure. If this first nucleotide is an adenosine, PCIF1 can deposit an additional methyl group at the N6 position to create 5´-m⁶Am cap. Finally, CMTR2 methylates the 2′-O position of the second transcribed nucleotide to produce cap 2 structure. Enzymes responsible for each catalytic step are shown in circles. Methyl groups are indicated by red squares

The discrimination between self and non-self RNA relies heavily on the specific enzymatic modifications of the 5′ end. The canonical m⁷G cap (cap 0) consists of a guanosine methylated at the N7 position and linked to the mRNA 5′ end via a 5′–5′ triphosphate bridge. Its formation requires coordinated enzymatic activity: first, RNA guanylyltransferase and 5′-phosphatase (RNGTT) removes the γ-phosphate from the 5′-ppp pre-mRNA and adds a guanosine monophosphate to create the triphosphate linkage. Subsequently, RNA guanine-7 methyltransferase (RNMT), together with its activator RAM, methylates the N7 position to generate cap 0 [20]. Following this, the mRNA can undergo 2′-O-methylation. The first nucleotide (N1) is methylated by cap methyltransferase 1 (CMTR1)—which is recruited by RNA polymerase II—to form cap 1. A second methylation at the second nucleotide (N2) is catalysed in the cytoplasm by CMTR2, forming cap 2 [21, 22]. Finally, if N1 is an adenosine, it can undergo N6-methylation by phosphorylated CTD interacting factor 1 (PCIF1) after 2′-O-methylation. This produces the N6,2-O-dimethyladenosine (m⁶Am) modification, which is abundant as the first transcribed nucleotide in eukaryotic mRNAs and serves as a refined marker of “self” (Fig. 2) [23].

### Sensing non-canonical motifs

IFIT proteins detect viral RNAs by recognising structural features that distinguish them from the canonical 5′-end architecture of host transcripts. IFIT5 binds with high affinity to RNAs terminating in a 5′-ppp, 5′-pp, or monophosphate (5′-p), largely regardless of sequence [10, 12]. Magnesium ions play a critical role in this interaction, facilitating IFIT5 association with RNAs bearing nicotinamide adenine dinucleotide (NAD⁺/NADH) caps—non-canonical modifications whose abundance is influenced by cellular metabolic state [24]. Although non-canonical caps such as flavin adenine dinucleotide (FAD) are more frequently associated with prokaryotic RNAs, they are also detected in eukaryotic and viral RNAs [25]. For example, the RNA-dependent RNA polymerase (RdRp) of Hepatitis C virus (HCV) can utilise FAD as a non-canonical initiating nucleotide. Importantly, while 5′-FAD capping protects RNA from RIG-I-mediated innate immune sensing, it does not confer protection against IFIT1 recognition [26–28]. In addition, IFIT1 can recognise 2,2,7-trimethylguanosine (TMG) caps present on small nuclear RNAs (snRNAs) [28]. By contrast, the metabolite diadenosine tetraphosphate (Ap4A), which is present in most eukaryotic cells, does not trigger a detectable response from any IFIT family member [29]. Collectively, these findings indicate that IFIT proteins function not only as antiviral sensors but also as modulators of endogenous RNA metabolism.

### Sensing methylation states

IFIT1 fulfils a specialised function in discriminating methylation states within canonical 5′ cap structures. It is the only IFIT family member known to selectively recognise cap 0 RNAs, which lack 2′-O-methylation at the first transcribed nucleotide, irrespective of nucleotide identity [24, 30]. This recognition is significantly potentiated upon complex formation with IFIT3 or within the IFIT1–IFIT2–IFIT3 heterotrimer. IFIT3 stabilises IFIT1, increasing its steady-state protein levels, and induces allosteric rearrangements within the RNA-binding channel. These rearrangements enhance the selective engagement of cap 0 RNAs while restricting binding to cap 1-modified transcripts [31, 32]. While earlier models suggested that the IFIT1–IFIT3 heterodimer represented the predominant and functionally most relevant configuration for antiviral surveillance, recent evidence reveals a more modular interactome. Importantly, the host also deploys methylation-independent sensing mechanisms; for instance, the IFIT2–IFIT3 complex can assemble independently of IFIT1 to restrict specific viruses [33], a mechanism detailed further below.

Host cells exploit differential cap methylation as a mechanism of transcriptome protection. The 2′-O-methyltransferase CMTR1 deposits the cap 1 modification on ISG mRNAs, thereby preventing their unintended translational repression by IFIT1 during the interferon response. Consistently, CMTR1 inhibition increases cellular permissiveness to Dengue virus (DENV) and Zika virus (ZIKV) replication, even though these flaviviruses encode their own viral methyltransferases capable of generating cap 1 structures to evade IFIT1-mediated restriction [34–36]. These findings suggest that the restriction of flaviviruses relies heavily on the stability of the host antiviral state—specifically the methylation of host ISGs by CMTR1—rather than solely on the capping status of the viral genome itself. Further experiments are required to fully elucidate the interplay between host mRNA methylation stability and viral replication dynamics.

An additional layer of regulation is conferred by CMTR2, which catalyses 2′-O-methylation of the second transcribed nucleotide to generate cap 2 structures. This modification is largely restricted to higher eukaryotes and is preferentially associated with long-lived mRNAs, contributing to transcript stability and efficient nuclear export [37–39]. Loss of CMTR2 activity leads to the accumulation of cap 1 RNAs that can engage RIG-I-dependent sensing pathways, resulting in heightened induction of interferon-stimulated genes, including IFIT1. Viral pathogens have adapted to this additional level of host discrimination. For instances, vesicular stomatitis virus (VSV) synthesises cap 1 structures via its multifunctional viral Large (L) polymerase but additionally exploits host CMTR2 to acquire cap 2 modifications on its viral transcripts [40]. This viral adaptability is likely a prerequisite for maximising the evasion of host innate immune recognition, thereby facilitating efficient viral replication.

### Sensing methylation-independent transcripts

In contrast to the methylation-dependent mechanisms of IFIT1, recent studies have expanded our understanding of the IFIT complex by demonstrating that IFIT2 and IFIT3 can structurally form an independent complex in the absence of IFIT1. The IFIT2–IFIT3 heterodimer functions as a molecular sensor that targets viral RNAs with short 5′ untranslated regions (UTRs) of less than 50 nucleotides (nt), binding to sequences proximal to the start codon to efficiently suppress translation. This mechanism highlights a critical front in the host-pathogen evolutionary arms race. Small, non-segmented negative-sense RNA viruses—such as VSV, rabies virus (RABV), and human parainfluenza virus type 3 (PIV3)—are evolutionarily constrained by limited genome sizes, forcing them to encode highly truncated 5′ UTRs to maximise available protein-coding space. The IFIT2–IFIT3 complex exploits this strict evolutionary constraint, utilising the short 5′ UTR as a structural PAMP, thereby restricting viral replication [33].

### m⁶Am: the stringent definition of self

The incorporation of m⁶Am into cap 1– or cap 2–containing RNAs establishes a stringent molecular definition of self. Biochemical assays demonstrate that 5′-m⁶Am caps are poorly recognised by IFIT proteins, with even the IFIT1–IFIT2–IFIT3 complex exhibiting limited binding affinity [31]. Consequently, the presence of m⁶Am enhances protein expression and attenuates type I interferon activation [28, 37]. Although the prevalence of 2′-O-methylation varies across tissues, m⁶Am remains a predominant feature of the mammalian 5′ cap. In contrast, cap-quantification studies indicate that this modification is absent from viral genomes such as DENV, which lacks the enzymatic machinery required to deposit m⁶Am [41, 42].Collectively, these findings support the designation of the 5′-m⁶Am cap as a defining molecular hallmark of self, underscoring the importance of elucidating its function for a comprehensive understanding of antiviral defence.

## Structural basis of 5´-RNA recognition

IFIT proteins are primarily localised in the cytoplasm and lack intrinsic catalytic activity. Their defining characteristic is the presence of multiple tetratricopeptide repeat (TPR) motifs; structural elements also found in many other host proteins. Each TPR consists of approximately 34 amino acids arranged in a helix–turn–helix configuration, enabling specific protein–protein interactions [43]. Although this fold is conserved, only nine residues at defined positions show limited conservation, leading to considerable variation in the primary sequences of TPRs both within a single IFIT protein and across the family. In humans, for instance, IFIT1 shares only 44% sequence identity with IFIT2 and IFIT3. This divergence is reflected in their structural composition: IFIT1 contains nine TPR motifs, whereas IFIT2 has four. Notably, IFIT2 also features a positively charged C-terminal region that enhances binding to RNA with AU-rich elements; mutations or deletions of charged residues in this region disrupt viral RNA interaction, thereby impairing its antiviral effectiveness against Newcastle disease virus [44]. IFIT proteins can specifically recognise viral RNA molecules with a 5′-ppp group or those lacking 2′-O-methylation at the 5′ end. This function is primarily mediated by IFIT1 and IFIT5 [10]. A detailed analysis of the structural features of these proteins provides critical insights into their distinct mechanisms of RNA recognition.

Structurally, IFIT1 comprises 23 α-helices, including nine TPRs that form a positively charged binding pocket. This site recognises viral RNAs with a 5′-ppp or lacking cap 1 structure, accommodating the N^7^-methylguanosine of cap 0, the triphosphate bridge, and up to four nucleotides [10]. Recognition involves specific residues—L46, L150, R187, I183, and Y218—while the “cap-binding loop” (T48, L46) positions the N^7^-methylguanosine. Phosphate groups are stabilised by K151, R187, R255, and R38, with additional inter-residue contacts (D34–R38, E176–W147) reinforcing binding. Notably, R38, K151, and R187 are particularly important as they surround the triphosphate bridge and accommodate the N^7^-methylguanosine of cap 0; mutations at these sites have been shown to disrupt RNA binding [11, 45].

IFIT5, in comparison, consists of 24 α-helices, including 18 TPR motifs arranged into three subdomains (I–III) together with a clamp-like pivot domain. IFIT5 does not bind canonical capped RNAs but instead recognises uncapped 5′-ppp-RNA via a positively charged channel. Key interactions occur at the channel (Q41, E33, R37, R186) and within subdomain II (T250, A253, K150). The coordination of a magnesium ion within the binding pocket masks the charge of E33, thereby anchoring interactions with the α/γ phosphates, while R186 establishes transient electrostatic contacts with the α- and β-phosphates [9,^10^,^11^, ^46^, ^47^]. The RNA substrate is positioned such that the first two nucleotides are buried within the binding tunnel, while downstream nucleotides remain solvent-exposed and interact with R260, K257, Q288, and R294. Mutational analyses confirmed that residues R186 and K150 are critical for RNA binding, although E33 appears to be dispensable. Consistent with this broad substrate recognition, IFIT5 exhibits minimal base specificity, enabling the recognition of structurally diverse viral RNAs bearing 5´-ppp termini [9–12]

## The arms race of 5´-RNA

Members of the IFIT family are strongly induced upon viral infection and have been shown across multiple species to interfere with viral translation by directly recognising viral 5′ cap RNAs in a conformation- and modification-dependent, but sequence-independent, manner [45, 48]. Despite the diverse and sophisticated countermeasures employed by viruses to mask these signatures, IFIT proteins continue to exert potent antiviral effects, as summarised in Table 1.

**Table 1:** Evolutionary divergence of IFIT-mediated antiviral restriction and viral evasion strategies

Virus Family	Virus	IFIT protein	IFIT Host Species	Target RNA & Restriction Mechanism	Viral Evasion / Capping Strategy	References
***Rhabdoviridae***	VSV	IFIT1	*Homo sapiens*	**Sequestration**: Direct binding of 5′-ppp RNA leader/mRNA; translation inhibition.	**Cap 1**: Synthesised by viral **L polymerase**.	[45]
		IFIT1	*Orcinus orca*	**Antiviral**: Reduction of viral infection		[14]
		IFIT5	*Pteropus alecto*	**Antiviral**: Recognition of 5’-ppp RNA; reduction of viral titre.		[49]
	VHSV	IFIT5	*Oncorhynchus mykiss*	**Antiviral**: Reduction of viral RNA transcripts.		[50]
***Orthomyxoviridae***	IAV	IFIT5	*Gallus gallus domesticus*	**Sequestration**: IFIT5 binds 5′-ppp;	**Cap-Snatching**: Cleavage of host capped mRNAs.	[51]
		IFIT1, 2	*Homo sapiens*	**Antiviral**: Reduction of viral infection		[52]
	IAV	IFIT5	*Pteropus alecto*	**Antiviral**: Reduction of H17N10 polymerase activity.		[49]
	IAV	IFIT5	*Anas platyrhynchos*	**Antiviral**: Reduction of H5N1 viral titre by enhancing IFN pathway		[53]
***Flaviviridae***	WNV ZIKV	IFIT1, 2	*Mus musculus*	**Cap Binding**: IFIT1 binds unmethylated Cap 0 (in viral NS5 mutants).	**2′-O-Methylation**: **NS5** MTase mimics Cap 1.	[35, 54]
	HCV	IFIT1	*Homo sapiens*	**Translation Inhibition**: Binds eIF3e to disrupt IRES initiation.	**IRES and** **FAD-Capping RNA**: Cap-independent translation.	[27, 55]
	DTMUV	IFIT5	*Anas platyrhynchos*	**Antiviral**: Reduction of viral titre by enhancing IFN pathway	**2′-O-Methylation**: **NS5** MTase mimics Cap 1	[56]
***Togaviridae***	VEE	IFIT1	*Homo sapiens*	**Structural Recognition**: Binds 5′ cap when stem-loop is destabilised.	**Cap 0**: Synthesised by viral **nsP1**. **Structural Shielding**: 5’ Stem-loop hides Cap 0.	[57]
		IFIT1	*Manis pentadactyla*	**Antiviral**: Reduction of viral infection		[14]
	SINV	IFIT1	*Orcinus orca / Mus musculus*	**Antiviral**: Reduction of viral infection	**Cap 0**: Synthesised by viral **nsP1**. **Structural Shielding**: 5’ Stem-loop hides Cap 0.	[14, 58]
***Paramyxoviridae***	PIV3	IFIT1	*Elephantulus edwardii*	**Antiviral**: Reduction of viral infection	**Cap 0**: Synthesised by viral **L polymerase**.	[14]
	RSV	IFIT1-3	*Homo sapiens*	**Indirect antiviral activity**: IAV-induced upregulation of IFIT proteins restricts secondary RSV replication during co-infection.		[59]
	SeV	IFIT5	*Homo sapiens*	**Antiviral**: Reduction of viral infection by enhancing IFN pathway		[60]
***Picornaviridae***	SVA	IFIT3	*Sus scrofa*	**Antiviral**: Interferes with multiple life cycle stages.	**IRES / VPg**: Cap-independent translation.	[61]
	FMDV	IFIT5	*Anas platyrhynchos*	**Antiviral**: Reduction of viral titre.		[35]
	EMCV	IFIT3	*Homo sapiens*	**Antiviral**: Reduction of viral infection by enhancing the IFN I		[62]
***Caliciviridae***	MNV	IFIT1	*Mus musculus*	**Antiviral**: Reduction of viral infection by enhancing the IFN I	**VPg**: Viral genome-linked protein.	[63]
***Birnaviridae***	IPNV	IFIT5	*Oncorhynchus mykiss*	**Antiviral**: Reduction of viral RNA transcripts.	**VPg**: Viral genome-linked protein.	[50]

Bold text indicates virus families, key host restriction mechanisms, and specific viral capping strategies or enzymes. Abbreviations: VSV, Vesicular Stomatitis Virus; Viral hemorrhagic septicemia virus, VHSV; Influenza A virus, IAV; West Nile virus, WNV; Zika virus, ZIKV; Hepatitis C virus, HCV; Duck Tembusu virus, DTMUV; Venezuelan equine encephalitis, VEE; Sindbis virus, SINV; Human Parainfluenza Virus Type 3, PIV3; Respiratory Syncytial Virus, RSV; Sendai virus, SeV; Senecavirus A, SVA; Foot-and-Mouth Disease Virus, FMDV; Encephalomyocarditis virus, EMCV; Murine norovirus, MNV; Infectious pancreatic necrosis virus, IPNV

### Mechanisms of translational inhibition

In both humans and mice, IFIT1 and IFIT2 inhibit cap-dependent viral translation by interacting with subunits of the eukaryotic initiation factor (eIF) 3 complex. This association disrupts the formation of the pre-initiation complex, which consists of the 40 S ribosomal subunit, eIF3, eIF2 bound to GTP and Met-tRNAi, together with eukaryotic initiation factor 4 F (eIF4F) [64]. As a result, recruitment of viral mRNAs to ribosomes is hindered, thereby blocking the initiation of protein synthesis. Importantly, eIF3 plays a crucial role not only in the translation of host capped mRNAs but also in that of certain viral RNAs, such as those of flaviviruses and coronaviruses that possess their own methyltransferases (MTases) [65, 66]. In addition, the eIF3 complex is required for the translation of viruses that utilise internal ribosome entry sites (IRESs), such as hepatitis C virus (HCV) and encephalomyocarditis virus (EMCV). IFIT1 has been shown to strongly inhibit translation of these viruses by binding to the eIF3e subunit, preventing the proper assembly of initiation complexes on the HCV IRES [55].

### Recognition of 5′-triphosphate RNA

A key mechanism of restriction involves the binding and sequestration of 5′-ppp RNAs, a hallmark of many viral genomes. For example, replication of VSV, which produces 5′-ppp subgenomic and leader RNAs, is markedly suppressed upon expression of IFIT1, IFIT2, and IFIT3 in host cells. A similar IFIT-dependent restriction has been reported for Rift Valley fever virus (RVFV) [45]. Viruses lacking an intrinsic capping machinery are therefore particularly susceptible. IFIT5 is unique among IFIT family members in its ability to specifically recognise these 5′-ppp RNAs. Accordingly, IFIT5 has been proposed to restrict the replication of negative-sense RNA viruses that lack proteins capable of generating capped RNAs, including influenza A virus (IAV) [50, 51, 67].

### Enzymatic mimicry

As part of an ongoing evolutionary arms race, cytoplasm-replicating RNA viruses—which cannot exploit the nuclear capping machinery—have evolved their own MTases to catalyse N-7 and 2′-O methylation of their RNA caps. For example, in flaviviruses, the NS5 protein carries an MTase domain that generates the characteristic cap 1 structure [37], whereas coronaviruses rely on the Nsp10/16 heterodimer to achieve cap 1 formation [68]. Although IFIT1, often in complex with IFIT2 or IFIT3, can recognise cap 1 structures in cells, the presence of 2′-O methylation determines whether these antiviral proteins can restrict viral replication [32]. This is exemplified by flaviviruses such as DENV, where proper 2′-O methylation of viral RNA promotes effective evasion of IFIT-mediated restriction [69].

### Cap-snatching and modification

Some viruses, such as orthomyxoviruses, have evolved mechanisms to protect their 5′-ppp RNAs by hijacking caps from cellular mRNAs. This process, known as “cap-snatching,” generates viral transcripts resembling cap 0, cap 1, or cap 2 structures and depends on the intracellular availability of host capped RNAs [70]. In the case of IAV, viral replication is reduced only in the presence of functional IFIT1, which can directly bind viral 5′-ppp RNA [52]. However, studies in murine models indicate that IAV replication is not significantly restricted by IFIT1, a phenomenon that also extends to other members of the *Bunyaviridae* and *Filoviridae* families [71]. Similarly, in human lung A549 cells, IFIT1 does not markedly impair IAV replication, likely due to cap-snatching from host mRNAs, which enriches viral transcripts with 2′-O-methylated caps (cap 1 and cap 2) [70, 71]. More recent evidence, however, demonstrates that IFIT2 and IFIT3 can independently bind IAV RNAs, thereby contributing to the restriction of IAV infection [72, 73].

Host cells have countered this strategy by evolving additional m⁶Am-modified caps, which enhance self-RNA recognition and are preferentially discriminated by IFIT1 [28, 37]. Intriguingly, several negative-sense RNA viruses—including RABV, VSV, and measles virus—subvert this defence by hijacking the host PCIF1 enzyme to deposit m⁶Am on their RNAs, thereby dampening IFN-β signalling and weakening the antiviral response [74].

### Structural shielding

Finally, some viruses lack the capacity to perform N-7 and 2′-O methylation yet still evade IFIT1 restriction. For instance, picornaviruses such as EMCV conceal the 5′ end of their genome with a covalently attached VPg protein, preventing IFIT recognition [75]. Similarly, although alphaviruses do not encode 2′-O-methyltransferases and would be expected to be highly sensitive to IFIT1, they are less affected than predicted. VEEV uses a stem–loop structure near the 5′ end of its RNA to shield the cap. A single nucleotide substitution at the third genomic position (G3→A) destabilises this structure, allowing IFIT1 to bind the cap directly and inhibit translation [57]. This strategy is also observed in Sindbis virus (SINV) [58], underscoring the strong evolutionary pressure imposed by IFIT1 on alphaviruses.

## Antiviral signalling

**Fig. 3:**
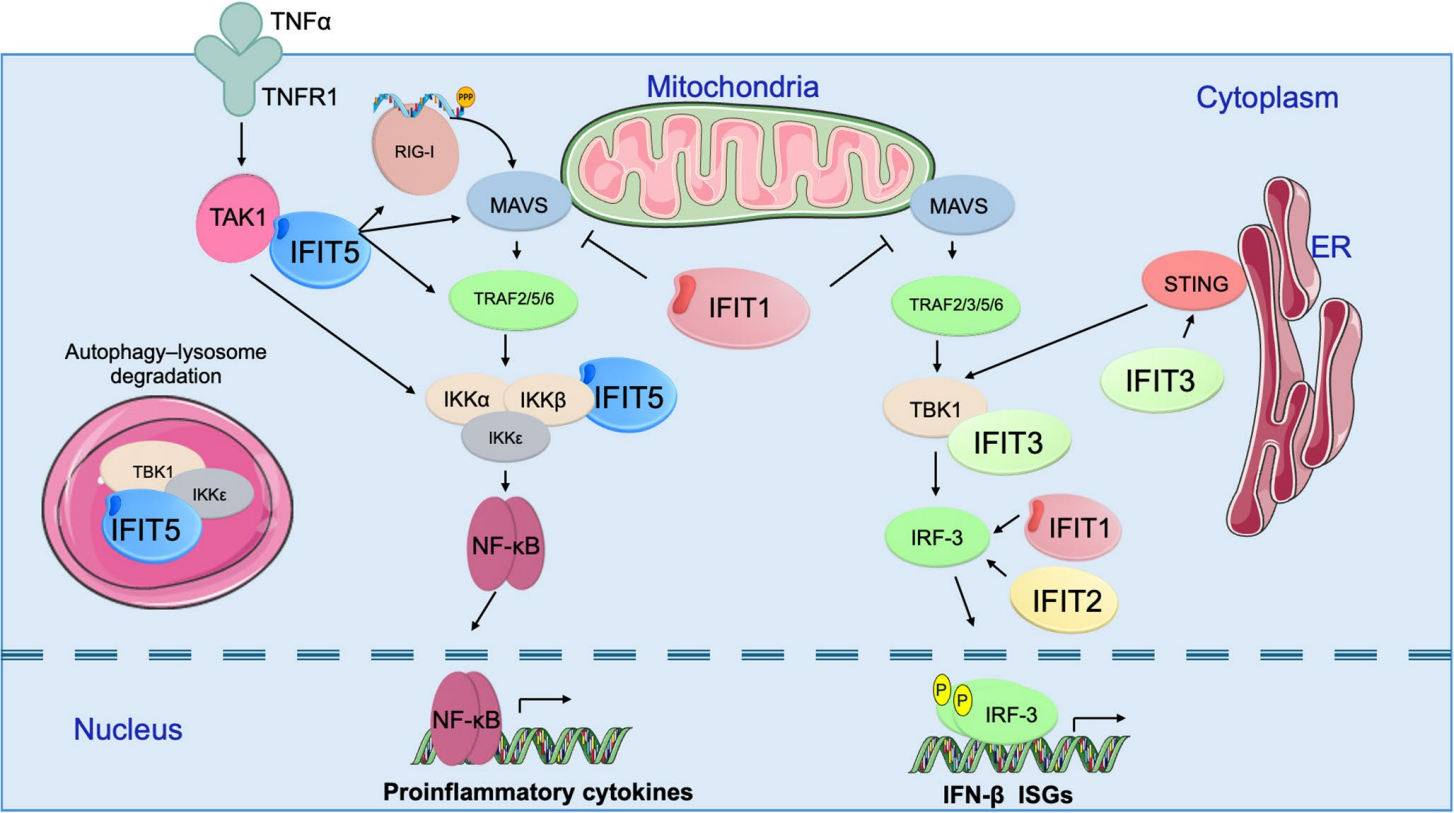
IFIT proteins function as versatile scaffolds in antiviral and inflammatory signalling cascades. IFIT proteins modulate two principal signalling pathways—NF-κB and IRF3—thereby regulating the transcription of pro-inflammatory cytokines and the induction of IFN-β–stimulated antiviral effectors, including ISGs. IFIT5 acts as a central modulator of NF-κB signalling through direct interactions with TAK1 and IKKβ, and by influencing upstream components of the RIG-I–MAVS–TRAF6 axis, thereby promoting pro-inflammatory cytokine production. In contrast, IFIT5 mediates the autophagic and lysosomal degradation of TBK1 and IKKε, leading to suppression of IRF3-dependent IFN-β expression. IFIT1 negatively regulates both NF-κB and IRF3 pathways by inhibiting MAVS signalling. However, IFIT family members can also enhance IRF3 activation. For instance, IFIT1 and IFIT2 promote IRF3 phosphorylation and nuclear translocation. IFIT3 interacts with TBK1 and positively regulates STING signalling, thereby promoting IFN-β production and ISG expression

Although basal expression of IFIT proteins remains low, they are rapidly upregulated in response to viral infection, IFN stimulation, or PAMPs such as dsRNA and lipopolysaccharides (LPS) [16, 76, 77]. Beyond their direct RNA-binding capabilities, IFIT proteins function as versatile signalling scaffolds. Their structural architecture is defined by multiple TPRs forming helix–turn–helix motifs, which mediate diverse protein–protein interactions [45]. Through these interactions, IFITs exert broad effects on antiviral immune responses and innate immunity, often functioning as critical nodes in signalling cascades (Fig. 3).

A clear example of positive regulation is observed with IFIT3, where its overexpression enhances—and its knockdown significantly reduces—antiviral gene expression. This potentiation is mediated through its interaction with TBK1 (TANK-binding kinase 1), a key adaptor in the MAVS signalling pathway [78]. Furthermore, IFIT3 has been reported to promote IFN-β activation via both the STING and MAVS pathways, acting as a bridge between cytosolic DNA and RNA sensing mechanisms [79]. Interestingly, the evolutionary role of IFIT3 extends beyond acting as a signalling adaptor or a cap-binding protein. For instance, it exerts direct antiviral activity against Senecavirus A (SVA), a single-stranded, positive-sense RNA virus, by interfering with multiple stages of the viral life cycle. Specifically, IFIT3 inhibits viral adsorption and internalisation, rather than directly blocking viral RNA replication [61]. These findings indicate that IFIT3 functions not only in amplifying antiviral signalling but also in modulating distinct, early phases of viral infection independent of direct RNA recognition.

In contrast, IFIT1 exhibits a complex, context-dependent function in regulating type I IFN and pro-inflammatory responses [80]. In human monocytes exposed to LPS, IFIT1 modifies the expression of interferon and inflammatory genes by modulating the Sin3A–HDAC2 transcriptional regulatory complex. Through this mechanism, IFIT1 cooperates with chromatin modulators to promote IRF3 recruitment at ISG loci, thereby optimising the innate immune response [81]. However, this regulatory profile shifts markedly in the context of viral infection. During Sendai virus infection, IFIT1 interacts with components of the MAVS pathway to inhibit the activation of IRF3, NF-κB, and IFN-β, effectively acting as a negative feedback regulator to restrict excessive inflammation [82]. Taken together, these observations suggest that IFIT1 exerts a dual role—promoting transcription in antibacterial (LPS) contexts while dampening signalling in specific viral contexts—though the precise determinants of this switch remain to be fully elucidated.

IFIT2 also acts as a critical intermediate in type I IFN signalling, particularly following TLR4 activation. Although initial reports in LPS-stimulated macrophages suggested that IFIT2 destabilises the mRNAs of pro-inflammatory cytokines such as IL-6 and TNF-α, this instability does not compromise cytokine secretion in vivo [83]. On the contrary, Ifit2 expression is essential for the systemic release of these cytokines; in Ifit2-deficient mice, serum levels of IL-6 and TNF-α are significantly reduced despite normal mRNA levels. This phenotype arises because IFIT2 is required to upregulate IRF3 phosphorylation, thereby enabling the type I IFN signalling loop [84]. The critical nature of this regulatory function is underscored in viral infection models: Ifit2-deficient neurons and immune cells exhibit significantly increased West Nile virus (WNV) and coronavirus replication due to impaired IFN-α/β expression, confirming that IFIT2’s antiviral potency extends beyond direct RNA binding to the amplification of host innate immunity [54, 85].

IFIT5 functions as an essential modulator of NF-κB and IRF3 signalling, with outcomes that depend heavily on the cellular context. Induced by viral stimuli (including RNA viruses and poly(I: C)), IFIT5 enhances inflammatory gene expression by interacting with MAVS signalosome components—including MAVS, RIG-I, and TRAF6—and directly binding to TAK1 and IKKβ. This interaction promotes IKK complex phosphorylation, accelerating the canonical activation of NF-κB and subsequent TNF-α production [60, 86]. This pro-inflammatory activity is subject to viral antagonism. A non-structural protein of SARS-CoV-2 has been reported to interact directly with IFIT5, promoting its degradation and consequently attenuating NF-κB–mediated inflammatory signalling. This mechanism enables viral evasion of innate immune activation [87].

Conversely, IFIT5 can exert inhibitory effects on type I interferon signalling. In certain viral contexts, IFIT5 promotes autophagy–lysosomal degradation of TBK1 and IKKε, thereby limiting IRF3 activation and reducing IFN-β induction. Consequently, IFIT5 has been reported to enhance infection by certain positive-strand RNA viruses, such as goose astrovirus (GAstV). This proviral effect is thought to arise not from direct RNA recognition, but rather from the suppressive influence of IFIT5 on broader antiviral signalling pathways, potentially creating a cellular environment that favours viral replication [88, 89] Collectively, these findings underscore the functional duality of IFIT5, positioning it as a versatile signalling adaptor capable of integrating and modulating pro-inflammatory and antiviral pathways in a stimulus-dependent manner.

## Targeting 5′-ends as antiviral strategies

Amid the ongoing evolutionary arms race between viruses and host cells over the chemical signatures at RNA 5′ ends, substantial progress has been made in combating viral infections by therapeutically exploiting IFIT activation. Considerable effort has focused on disrupting viral 2′-O-methylation, thereby preventing the formation of cap 1 structures and increasing the susceptibility of viral RNAs to IFIT-mediated recognition. These approaches include the development of small-molecule inhibitors targeting viral and host 2′-O-methyltransferases responsible for cap 1 deposition, most notably NS5 in flaviviruses, the nsp10/nsp16 complex in coronaviruses, and the host methyltransferase CMTR1 [32, 36, 90].

### Targeting flaviviruses

A particularly relevant target for evaluating these strategies is the NS5 protein of flaviviruses, a multifunctional enzyme that serves as an RNA-dependent RNA polymerase (RdRp) and possesses both N7-methyltransferase (N7-MTase) and 2′-O-methyltransferase (2′-O-MTase) activities. While these functions rely on distinct amino acid residues, both are essential for efficient viral replication. Specifically, mutations that abolish 2′-O-MTase activity prevent the formation of the cap 1 structure, thereby exposing the viral RNA to host surveillance. In the absence of this methylation, viral transcripts from WNV are rendered hypersensitive to recognition and sequestration by IFIT1 and IFIT2 [35, 91].

Consequently, these NS5 mutants exhibit severe replication defects and attenuation in vivo, a characteristic that has been exploited for vaccine development. Indeed, DENV serotypes 1 and 2, as well as Japanese encephalitis virus (JEV) strains harbouring engineered MTase mutations, have been proposed as live-attenuated vaccine candidates. These variants confer strong protection against subsequent challenge in animal models precisely because they induce a robust IFN-β response, thereby promoting immunogenicity while preventing pathogenicity [90, 92].

Beyond genetic attenuation, pharmacological inhibition of NS5 is a major focus. Although the residues responsible for 2′-O-MTase activity are positioned differently among distinct flaviviruses—posing a challenge for broad-spectrum drug development—high-throughput screening has identified candidate inhibitors capable of suppressing the replication of WNV, DENV2, and Yellow fever virus (YFV). Notably, several of these compounds, such as the nucleoside analogues GRL-002 and GRL-003, inhibit WNV replication by targeting both the N7-MTase and 2′-O-MTase activities of NS5 without inhibiting human RNMT [93, 94]. This specificity reinforces the feasibility of developing MTase-selective antiviral agents that spare host enzymes.

### Targeting coronaviruses

The nsp10/nsp16 Complex Parallel efforts have targeted the coronavirus nsp10/nsp16 complex, a viral methyltransferase that utilises S-adenosylmethionine (SAM) as the methyl donor for RNA cap modification. In silico screening efforts have predominantly focused on the SAM-binding pocket and the nsp10–nsp16 interaction interface [95–97], as disruption of either region compromises 2′-O-methylation and renders viral RNAs susceptible to IFIT restriction. For instance, Tubercidin was identified through virtual screening as an inhibitor of nsp16 2′-O-methyltransferase activity, resulting in strong suppression of SARS-CoV, MERS-CoV, and SARS-CoV-2 replication in cell culture [98].

### Host-directed strategies interfering with cap-snatching

Notably, interference with 2′-O-methylation represents a broader antiviral strategy that extends beyond the direct inhibition of viral enzymes to include host-directed targets. During Influenza A virus (IAV) infection, the host enzyme CMTR1 plays a critical role in facilitating cap-snatching while simultaneously limiting innate immune detection. In this setting, the Streptomyces-derived compound trifluoromethyl-tubercidin (TFMT) has been shown to suppress IAV replication by disrupting the interaction between host capped RNAs and the viral polymerase basic protein 2 (PB2) subunit. Importantly, TFMT exhibits minimal cytotoxicity and retains antiviral activity in ex vivo models [99].

By contrast, TFMT displays limited activity against SARS-CoV-2, underscoring the specific dependence of IAV on host CMTR1. Interestingly, TFMT exhibits potent antiviral activity against avian coronavirus (infectious bronchitis virus, IBV), but fails to restrict other viruses such as Hazara virus (HAZV), which requires cap-snatching mechanisms for proper replication [99, 100]. This discrepancy indicates that the requirement for CMTR1 during viral replication is highly context dependent. It is probable that the reliance on host CMTR1 represents a host-specific evolutionary adaptation. In higher organisms, such as mammals, this host–virus co-evolution implicates the RNA 5′-end modification pathway as a critical vulnerability in RNA virus replication. Interfering with these capping and cap-snatching mechanisms—whether viral or host-derived—is likely to promote IFIT activation by exposing vulnerable 5′-end RNA signatures that are normally concealed during replication. However, further experimental work is required to define the precise molecular mechanisms through which cap disruption engages IFIT-mediated restriction across diverse viral contexts.

### Concluding remarks

The ongoing molecular arms race between host innate immunity and viral replication is centred on the precise discrimination between self and non-self RNA. As detailed in this review, IFIT proteins have evolved as specialised sensors that exploit structural and chemical signatures at the RNA 5′ end—specifically the absence of 2′-O-methylation or the presence of a 5′-ppp—to selectively inhibit viral translation. However, the remarkable plasticity of viral genomes has driven the emergence of diverse evasion strategies, ranging from the recruitment of host methyltransferases to cap-snatching mechanisms that mimic host-specific modifications such as cap 1, cap 2, and m⁶Am. The influence of cellular metabolism on non-canonical RNA capping (e.g., NAD⁺ and FAD) represents an additional and underexplored layer of host–pathogen interaction. Addressing these gaps in our understanding of the structural and functional interplay between host RNA-binding proteins and viral RNAs will be critical for anticipating viral evolutionary trajectories.

This viral plasticity has driven the evolution of a dual-layered surveillance system capable of discriminating between cap-dependent and cap-independent molecular signatures. While the roles of IFIT1 and IFIT5 as primary antiviral effectors—recognising 2′-O-methylation status and 5′-triphosphorylated (5′-ppp) RNA, respectively—are well established, the functions of IFIT2 and IFIT3 are emerging as a critical frontier in the field. Initially characterised for their allosteric regulation of IFIT1, including modulation of complex stability and RNA-binding affinity, recent evidence indicates that the IFIT2–IFIT3 complex can also function as an independent molecular sensor. Specifically, this complex recognises short 5′ UTRs (< 50 nt) of compact non-segmented RNA viral genomes as structural PAMPs, binding proximal to the start codon [33]. By targeting these structural constraints, the host immune system exploits an inherent viral vulnerability. Given that compact viral genomes must minimise 5′ UTR length to maximise coding capacity, this constraint becomes a functional liability that enables precise immune recognition.

Ultimately, elucidating these molecular interactions offers a renewed prospect for controlling viral infections. Future antiviral strategies need not be limited to inhibiting viral 2′-O-methyltransferases; there is significant therapeutic potential in targeting the host cellular machinery hijacked by viruses to facilitate their replication, particularly the enzymes responsible for generating host cap structures. Although viruses have historically evolved sophisticated mechanisms to evade IFIT surveillance, their obligate reliance on these specific host modifications remains a distinct evolutionary vulnerability. By pharmacologically disrupting these capping machineries, we can effectively abrogate the molecular mimicry of viral RNAs. Such interventions would re-sensitise viral transcripts to IFIT-mediated recognition, thereby turning the host’s innate restriction mechanisms back against the pathogen and reinforcing the barrier to cross-species transmission.

## Data Availability

No datasets were generated or analysed during the current study.
